# Factor Analysis of Semen Quality in Chicken and Its Impact on Fertility

**DOI:** 10.3390/ani15131906

**Published:** 2025-06-28

**Authors:** Yunlei Li, Yanyan Sun, Aixin Ni, Hailai Hagos Tesfay, Adamu Mani Isa, Yunhe Zong, Hui Ma, Jingwei Yuan, Jilan Chen

**Affiliations:** 1 State Key Laboratory of Animal Biotech Breeding, Institute of Animal Science, Chinese Academy of Agricultural Sciences, Beijing 100193, China; liyunlei@caas.cn (Y.L.); sunyanyan02@caas.cn (Y.S.); naixin951@163.com (A.N.); kibatunovel12@gmail.com (H.H.T.); isa.adamu@udusok.edu.ng (A.M.I.); zongyunhe2022@163.com (Y.Z.); mahui@caas.cn (H.M.); yuanjingwei@caas.cn (J.Y.); 2 Department of Animal Science, Usmanu Danfodiyo University, Sokoto 840104, Nigeria

**Keywords:** chicken, semen quality, CASA, factor analysis

## Abstract

Evaluation of semen quality is crucial for predicting fertility success in artificial insemination and eventual rooster selection within poultry breeding programs. However, the multitude of parameters obtained in semen quality analysis makes it challenging for breeders to make informed selection decisions. This study evaluated semen samples from 210 roosters in seven chicken breeds, using a computer-aided sperm analysis (CASA) system. A multivariate approach utilizing factor analysis (FA) was applied to simplify the data. The FA condensed 14 semen quality traits into 3 factors, linearity factor, velocity factor, and quantitative factor, explaining 75.82% of the original variations. The Beijing-You breed was selected to analyze the association between fertility and these semen quality factors, and we found high correlation between fertility and quantitative (r = 0.84) and linearity (r = 0.63) factors, and low correlation with the velocity factor (r = 0.19). Based on individual factor scores, breed clustering revealed distinct profiles with Houdan, Tibetan, and White Leghorn demonstrating lower sperm counts and poor linearity. Columbian Plymouth Rock exhibited high speed with poor linearity while Beijing-You, Rhode Island Red, and Barred Plymouth Rock displayed higher sperm counts with compromised speed and linearity. This study demonstrates that FA effectively consolidates multiple semen quality traits into fewer, biologically relevant factors. These findings provide valuable insights into breed-specific reproductive characteristics and offer actionable information for optimizing breeding in poultry production.

## Simple Summary

Evaluation of semen quality is crucial for predicting fertility success in artificial insemination and eventual rooster selection within poultry breeding programs. With modern technology and equipment such as computer-aided sperm analysis, we can obtain high dimensional semen quality data. However, the multitude of parameters obtained in semen quality analysis makes it challenging for breeders to make informed selection decisions. To obtain more meaningful results and better guide breeding practices, analytical methods need further improvement. Here, we examined the semen quality of 210 roosters from seven breeds and condensed 14 traits into 3 factors explaining 75.82% of the variation using factor analysis. The Beijing-You was selected to analyze the correlation coefficients between fertility and these semen quality factors, and we found high coefficients between fertility and quantitative (0.84) and linearity (0.63) factors, but much lower with the velocity factor (0.19). This study demonstrates that factor analysis effectively combines multiple semen quality traits into fewer, biologically relevant factors. These findings provide valuable insights into breed-specific reproductive characteristics and offer actionable information for optimizing breeding in poultry production.

## 1. Introduction

Assessing semen quality is essential for predicting fertilization ability of roosters, particularly within the context of artificial insemination (AI) and determining the recommended sperm dose per insemination [1,2]. Accurate assessment of fertilization ability is essential for breeding purposes, as various sperm characteristics are routinely examined to improve fertility outcomes. Routine semen quality traits such as semen volume, pH, and concentration are commonly evaluated, while computer-aided sperm analysis (CASA) provides objective measurements of sperm kinematic parameters in various species, including chickens [3,4].

However, relying solely on routine semen quality traits and CASA-derived kinematic parameters has limitations. While routine semen analysis is subjective and, therefore, prone to errors, it also makes detection of subtle variability between semen of different roosters difficult. CASA-derived metrics are detailed, repeatable, and offer valuable insights, but they are not entirely independent. Hence, univariate analysis may obscure underlying patterns within the data [5]. The complex relationships among variables can mask meaningful information when examined in isolation, particularly regarding how motion parameters collectively relate to fertility in chickens. It is necessary to identify a few comprehensive variables that can retain most of the original information, enabling dimensionality reduction and facilitating concise and effective statistical analysis.

Multivariate analysis techniques have been widely applied across various fields, such as meat quality [6,7], and morphological [8] and behavior [9] traits, and offer useful tools for analyzing correlated variables. Multivariate methods including principal component analysis (PCA) [10,11,12], cluster analysis (CA) [13], and Chernoff faces [14] have been used to analyze CASA-derived kinematic data. PCA aims to summarize datasets through linear combinations of original variables, while factor analysis (FA) reduces dimensionality by modeling and factor rotation to yield interpretable results [15]. These multivariate approaches provide effective tools for exploring complex semen quality data.

In this study, we evaluated semen quality of seven chicken breeds representing distinct contributions to poultry production—ranging from egg-laying to dual-purpose traits, color varieties, and local adaptability. Our objective was to reduce multiple semen quality traits into fewer, biologically relevant factors using multivariate approaches, and the Beijing-You breed was selected to examine the correlations between these factors and fertility. This work aimed to provide insights to guide poultry breeders in rooster selection.

## 2. Materials and Methods

### 2.1. Animals

**Figure 1:**
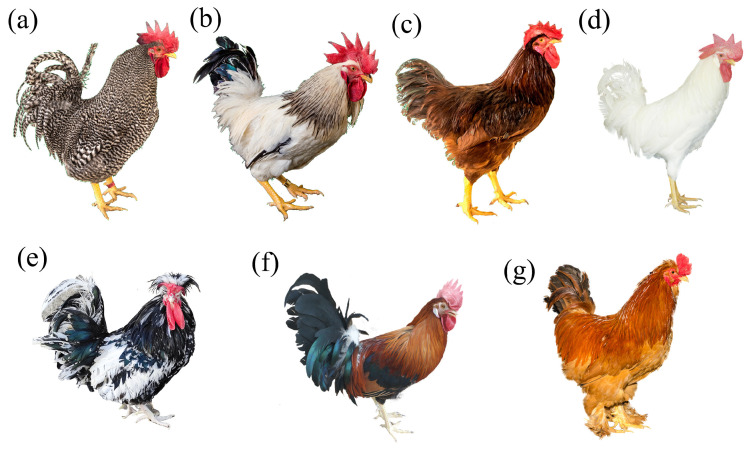
Images of Barred Plymouth Rock (**a**), Columbian Plymouth Rock (**b**), Rhode Island Red (**c**), White Leghorn (**d**), Houdan (**e**), Tibetan (**f**), and Beijing-You (**g**) roosters.

This study was conducted at the experimental farm of IAS-CAAS. A total of 210 roosters (50 weeks old at the onset of the experiment, 30 for each breed) were selected from Barred Plymouth Rock, Columbian Plymouth Rock, Rhode Island Red, White Leghorn, Houdan, Tibetan, and Beijing-You breeds as shown in Figure 1. Barred Plymouth Rock, Columbian Plymouth Rock, Rhode Island Red, and White Leghorn were pure breeds sourced from the University of Guelph, Canada. Beijing-You chickens were sourced from the National Beijing-You chicken conservation farm (IAS-CAAS) in Beijing, China. The Tibetan breed originated from a conservation farm in Lhasa City, Tibet Autonomous Region, China, while the Houdan breed was obtained from Guangdong Ocean University, Guangdong province, China.

One hundred Beijing-You hens were selected for AI to determine the individual fertility of ten Beijing-You roosters with a sex ratio of 1:10. All animals were healthy and free from any physical defects. The animals were kept in individual cages and maintained under a light regime of 16L:8D (light intensity of 20 lx), with lights off between 22:00 and 06:00 h. Animals had free access to a standard diet (18% crude protein) and water.

### 2.2. Semen Collection and Quality Assessment

The roosters were cleaned around cloacae and pre-trained to respond to abdominal massage technique [16] every other day for one week prior to the formal semen quality evaluation. The ejaculate was collected manually in a weighted micropipette, maintained at 37 °C, and taken for semen quality analysis within 10 min of collection. Contaminated ejaculates were discarded. Semen collection during the experiment was performed by the same skilled technician. Semen quality was estimated three times at a two-day interval. Semen color was evaluated immediately after collection in the transparent collection tube. Semen volume (VOL) was measured by a weighing method following World Health Organization (WHO) laboratory manuals [17]. Semen pH was determined using a pH meter (Seven Compact S210, Mettler-Toledo instruments Co., Ltd., Schwerzenbach, Switzerland). Sperm concentration (CON), sperm motility, and sperm kinematics parameters were estimated using CASA system (ML-608JZII; Nanning Songjingtianlun Bio-technology Co., Ltd., Nanning, China). Briefly, ten μL semen samples were diluted in 990 μL pre-warmed (37 °C) Dulbecco’s Modified Eagle’s Medium (DMEM, Gibico, C11995500BT, Beijing, China). After thorough mixing, ten μL of diluted semen was transferred to the sperm counting chamber (20 μm depth) placed on a pre-warmed (37 °C) microscope stage. Five fields in the chamber per sample were selected and captured by the CASA system from the microscope equipped with a 10× negative phase-contrast lens (ML-608JZII) for further analysis. During the analysis, the brightness and contrast of sperm sample images were adjusted to enhance their visibility for CASA, and the automatic analysis function of the system was employed to analyze the samples. CON, sperm motility, and sperm kinematic parameters including curvilinear velocity (VCL), average path velocity (VAP), straight line velocity (VSL), linearity (LIN, LIN = VSL/VCL × 100%), straightness of the average path (STR, STR = VSL/VAP × 100%), and wobble (WOB, WOB = VAP/VCL × 100%) were obtained. Total sperm count (TSC) and effective sperm count (ESC) were calculated for each rooster using the relationships as below:

TSC = semen volume × sperm concentration

ESC = TSC × sperm motility.

### 2.3. Fertility Analysis of Beijing-You Roosters

Ten Beijing-You roosters were selected, and their fertility was determined by inseminating hens with 50 μL of individual fresh semen obtained from each rooster. Eggs were collected within a 21-day post-insemination period, beginning on the next day after two consecutive inseminations. Each egg was marked to identify the sire and then incubated at 38.0 °C for five days. Fertility was assessed by examining contents for embryonic development on 5 days post-incubation via candling [18]. Briefly, a faint network of blood vessels with either a developing eye (healthy embryo) or a red ring (dead embryo) were counted as fertilized eggs. Clear eggs were counted as unfertilized.

### 2.4. Statistical Analysis

One-way analysis of variance (ANOVA) was performed to assess the statistical significance of differences among the seven breeds for each trait using PROC glm in SAS software (version 9.2; SAS Inst. Inc., Cary, NC, USA). Correlation analysis among semen quality variables was performed using PerformanceAnalytics package in R (version 4.0.2). The level of significance was set at *p* < 0.05. To evaluate the suitability of the FA, *Bartlett’s* test of sphericity and the Kaiser–Meyer–Olkin (KMO) test were carried out using SAS. FA was conducted using PROC factor in SAS, with PCA used for factor extraction. Varimax rotation was applied to ensure factor uniqueness and enhance interpretability. The regression method was utilized to predict factor scores based on the original semen quality variables.

## 3. Results

### 3.1. Analysis of Semen Quality Traits of Seven Breeds

The results of breed differences in semen quality traits are summarized in Table 1. Significant variations were observed among breeds for multiple parameters (*p* < 0.01). Average semen volume was significantly higher in Beijing-You, Barred Plymouth Rock, and Rhode Island Red compared to the White Leghorn, Houdan, and Tibetan breeds (*p* < 0.01). The Columbian Plymouth Rock breed exhibited the highest average semen concentration among all breeds (*p* < 0.01), but the overall mean concentration across breeds was 2.0 billion/mL. Sperm motility was significantly greater in Rhode Island Red, Barred Plymouth Rock, Columbian Plymouth Rock, and Beijing-You compared to the White Leghorn, Tibetan, and Houdan breeds (*p* < 0.01), but ranged from 42% to 70% across breeds. Beijing-You had the highest total sperm count, followed by Rhode Island Red, Barred Plymouth Rock, Columbian Plymouth Rock, Tibetan, Houdan, and White Leghorn. This trend was mirrored in the effective sperm count. Semen pH was significantly more alkaline in White Leghorn, Houdan, and Tibetan compared to Rhode Island Red, Barred Plymouth Rock and Columbian Rock (*p* < 0.01). The semen pH was neutral in Beijing-You, but semen pH ranged from 6.99 to 7.52 across breeds. The values of VCL, VSL, and VAP ranged from 50 and 60 μm/s, 22 and 29 μm/s, and 39 and 54 μm/s, respectively. Barred Plymouth Rock and Beijing-You had significantly lower VCL (*p* < 0.01) and Columbian Rock and White Leghorn had significantly higher VAP than other breeds. The Rhode Island Red, Barred Plymouth Rock, and Tibetan breeds showed higher VSL values (*p* < 0.01). Barred Plymouth Rock and Rhode Island Red displayed higher LIN and STR (*p* < 0.01) but lower BCF and WOB (*p* < 0.01). ALH was significantly lower in Barred Plymouth Rock and Beijing-You compared to other breeds (*p* < 0.01).

### 3.2. Correlation Analysis of Semen Quality Traits

**Figure 2:**
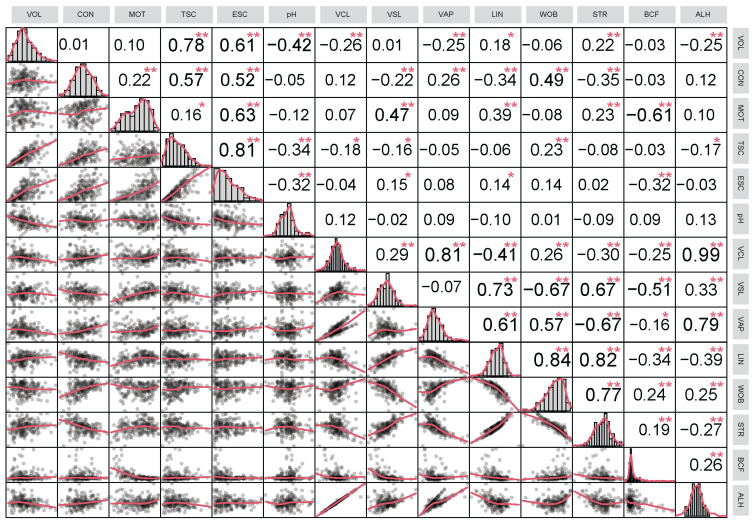
Correlation coefficients between semen quality traits. Note: The matrix chart (upper) summarizes the Pearson correlation. The scatterplot (lower) shows the relationship between two variables for the same individuals. The small bar graphs (diagonal) represent the distribution of data for each variable. VOL, semen volume; CON, sperm concentration; MOT, sperm motility; TSC, total sperm count; ESC, effective sperm count; VCL, curvilinear velocity; VSL, straight-line velocity; VAP, average path velocity; LIN, linearity; WOB, wobble; STR, straightness; ALH, amplitude of lateral head; BCF, beat cross frequency. * *p* < 0.05; ** *p* < 0.01.

Significant Pearson’s correlations were observed among the semen quality parameters, as shown in Figure 2. Sperm motility positively correlated with concentration (r = 0.22), VSL (r = 0.47), LIN (r = 0.39), and STR (r = 0.23), but negatively with BCF (r = −0.61). Both total and effective sperm counts showed positive relationships with volume, concentration, and motility, but negative correlations with pH. Semen volume was negatively correlated with pH (r = −0.42). Significant negative correlations were observed among different sperm kinematic parameters. VCL was positively correlated with VSL (r = 0.29), VAP (r = 0.81), WOB (r = 0.26), and ALH (r = 0.99), but negatively with LIN (r = −0.41), STR (r = −0.30), and BCF (r = −0.25). Furthermore, VSL was positively correlated with LIN (r = 0.73), STR (r = 0.67), and ALH (r = 0.33) but negatively with WOB (r = −0.67) and BCF (r = −0.51). VAP showed strong positive correlations with ALH (r = 0.79), LIN (r = 0.61), and moderate positive correlation with WOB (r = 0.57), but a negative correlation with STR (r = −0.67) and BCF (r = −0.16). Notable high positive correlations existed between STR and LIN (r = 0.82) and between STR and WOB (r = 0.77).

### 3.3. Factor Analysis of Semen Quality Traits

Bartlett’s test indicated a chi-squared value of 4038.38 (*p* < 0.001) and a KMO value of 0.65. The first three factors explained 31.34%, 23.28%, and 21.19% of the variance, respectively, cumulatively accounting for 75.82% (Table 2). Based on the rotated component matrix presented in Table 3, Factor 1 (F1) loaded highly on VSL (0.92), LIN (0.88), and STR (0.82), while showing strong negative loading for WOB (−0.82) and BCF (−0.58). Factor 2 (F2) showed high loading for VCL (0.93), ALH (0.93), and VAP (0.86), with a moderate negative loading on BCF (−0.47). Factor 3 (F3) was characterized by high loading on TSC (0.94), ESC (0.94), and VOL (0.78), alongside a negative loading on pH (−0.47).

### 3.4. Analysis of Semen Factor Scores Among Seven Breeds

**Figure 3:**
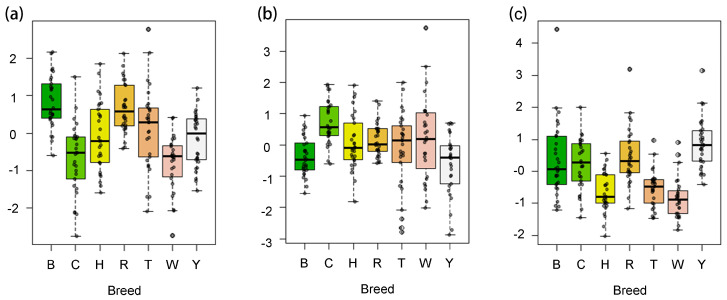
Box-plot of the factor scores in seven breeds. Note: (**a**) Factor 1 scores of seven breeds; (**b**), Factor 2 scores of seven breeds; (**c**), Factor 3 scores of seven breeds; B, Barred Plymouth Rock; C, Columbian Plymouth Rock; H, Houdan; R, Rhode Island Red; T, Tibetan; W, White Leghorn; Y, Beijing-You.

Predicted factor scores for semen quality traits in the study breeds are presented in Figure 3. The Houdan, Tibetan, and White Leghorn had lower F3 values (Figure 3c) while White Leghorn, Tibetan, and and Columbian Rock had lower F1 values (Figure 3a). Barred Plymouth Rock, Houdan, and Beijing-You had lower F2 scores (Figure 3b).

**Figure 4:**
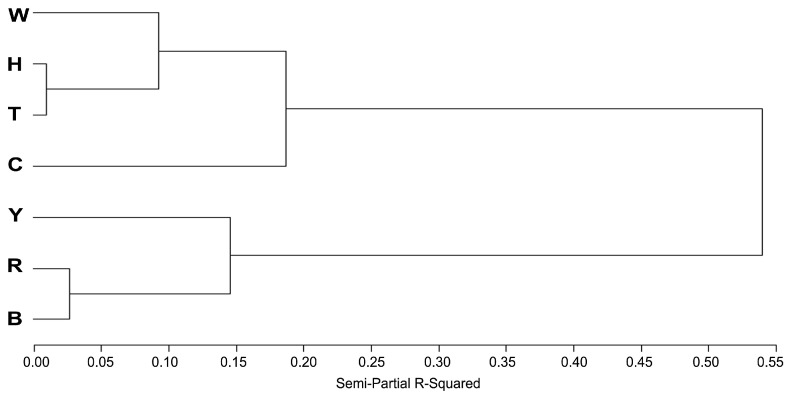
Hierarchical clustering of seven breeds based on predicted factor scores. Note: B, Barred Plymouth Rock; C, Columbian Plymouth Rock; H, Houdan; R, Rhode Island Red; T, Tibetan; W, White Leghorn; Y, Beijing-You.

Clustering of the seven breeds based the factor scores are presented in Figure 4. Houdan, Tibetan, and White Leghorn clustered together due to their low F3 and intermediate F1 and F2 scores. Rhode Island Red and Barred Plymouth Rock formed a distinct cluster with high F1 and intermediate F3 values. Beijing-You exhibited the highest score in F3 and lowest F2 score but clustered alongside Rhode Island Red and Barred Plymouth Rock breeds. Columbian Plymouth Rock was unique with its highest F2 score but low F1 score.

### 3.5. Correlation Among Fertility, Semen Quality Traits, and Their Factor Scores in Beijing-You

**Figure 5:**
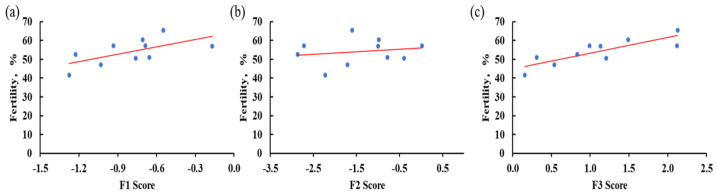
Scatterplots of the relationship between fertility and Factor 1 score (**a**), Factor 2 score (**b**), and Factor 3 score (**c**). Note: The red lines show the trend lines, blue circles represent individual samples according to factor scores and fertility values.

A total of 1120 settable eggs were collected during the experiment and 632 eggs were fertilized, yielding an average fertility of 56.43%. Significant Pearson’s correlations were observed between fertility and semen predicted factor scores, as shown in Figure 5. Results reveal a very strong positive correlation between fertility and the predicted Factor 3 score (r = 0.84, *p* = 0.03). Additionally, a significant strong positive correlation was observed with the predicted Factor 1 score (r = 0.63, *p* = 0.05). No significant association was detected between fertility and the predicted Factor 2 score (r = 0.19, *p* = 0.60). Pearson’s correlation between fertility and individual semen parameters including semen volume, sperm concentration, sperm motility, TSC, ESC, semen pH, VCL, VSL, VAP, LIN, WOB, STR, BCF, and ALH were 0.75 (*p* = 0.01), 0.17 (*p* = 0.63), 0.47 (*p* = 0.17), 0.69 (*p* = 0.03), 0.73 (*p* = 0.02), −0.32 (*p* = 0.37), 0.14 (*p* = 0.69), 0.35 (*p* = 0.32), 0.08 (*p* = 0.83), 0.40 (*p* = 0.26), −0.31 (*p* = 0.39), 0.21 (*p* = 0.55), −0.54 (*p* = 0.11), and 0.14 (*p* = 0.69), respectively.

## 4. Discussion

Semen analysis serves as a critical tool in evaluating male fertility potential, yet single parameter analysis cannot reliably predict fertility due to the interdependence of traits [19,20,21]. The results of the ANOVA confirm significant breed effects, revealing the genetic diversity among the breeds concerning each semen quality trait. Furthermore, the semen parameter values were consistent with previous findings [5,22,23], suggesting that new approaches for data analysis are necessary to mitigate information overlap and provide comprehensive assessments for practical applications. We, therefore, employed multivariate statistical approaches to analyze the interplay between semen quality traits and fertility in the seven chicken breeds. Our results demonstrate that factor analysis, specifically principal component analysis, proved effective in disentangling the complex relationships among multiple semen quality variables. This method facilitated a reduction of numerous indicators into three meaningful factors, explaining a substantial proportion (75.82%) of the total observed variability in the dataset.

Linearity factor termed F1, represents sperm motility and direction, such as progressive motility. Velocity factor (designated F2) is determined by variables related to overall movement speed but potentially less directional consistency, while F3, labeled as the quantitative factor, represents parameters like sperm concentration and volume. These predicted factors provided a powerful framework for characterizing breed-specific differences. Notably, F1 scores were highest in the Barred Plymouth Rock and Rhode Island Red breeds, suggesting a potential advantage related to sperm motility and direction in these breeds. Conversely, the Columbian Plymouth Rock showed prominence in F2, potentially reflecting unique selection histories for sperm speed or unintended consequences of breeding practices [24]. The Beijing-You strain demonstrated superior performance in F3, consistent with its documented history of selective breeding for enhanced semen volume and sperm motility [25,26]. In contrast, the White Leghorn, Tibetan, and Houdan breeds exhibited lower scores for Factor 3, highlighting a potential area for targeted genetic improvement to enhance overall semen volume and concentration.

The multivariate approaches demonstrate effective ability in assessing rooster fertility potential. This confirmed the findings of Agarwal et al. [5] who demonstrated the capacity of PCA to condense nine sperm parameters into two principal components they termed “semen quality” and “relative semen quality”, which, together, explained 80.4% of the total variability. They concluded that PCA offers an enhanced assessment of male fertility for clinician applications. In Egyptian buffalo bulls, PCA was successfully applied to reduce numerous semen variables across multiple ejaculates into a few principal components, facilitating the identification of distinct genetic groups based on semen traits [27]. Similarly, Wang et al. [28] applied FA to five semen traits, identifying factors termed “quantitative factor” and “quality factor”, suggesting potential for efficient evaluation. In this study, we observed significant Pearson’s correlations between fertility and semen predicted factor scores. Fertility significantly correlated positively and strongly with predicted F3, and moderately with F1. High scores for F3 indicate a higher sperm quantity, ensuring adequate sperm cells are delivered per insemination with equal volume of fresh semen. This aligns with previous reports that linked higher sperm count with increased fertility in roosters [29]. High scores for F1 indicated the ability of spermatozoa to swim in a relatively straight path. Interestingly, predicted F2 showed no significant association with fertility, indicating a limited contribution sexual potency of roosters in the current study. While F2 emphasizes velocity over linearity stressed by F1, these results suggest that effective fertilization for sperm likely depends more on directed movement than on speed alone [30]. Previous studies have shown that PCA could effectively classify animals with high or low sperm freezability based on post-thaw motility, viability, and sperm kinetic parameters in bulls [31] and rams [32]. The potential use of FA to evaluate other semen quality-related traits, such as freezing tolerance, requires further confirmation.

Single-parameter analyses highlight the importance of traits like semen volume and sperm motility, which have shown significant positive correlations with fertility [33,34], corroborating the findings of the current study. Previous studies linked CASA-derived parameters such as VSL, VCL, and LIN to fertility outcomes in humans [35] and animals [36].

Together, our findings highlight the predictive power of FA in assessing fertility of roosters from numerous semen variables. The significant correlations between fertility and predicted factor scores suggested that these integrated approaches capture complex relationships among semen parameters more effectively than individual traits alone in assessing fertility potential. This will simplify rooster selection through the reduction of multiple semen traits into key factors. These factor scores will allow breeders to rank individuals, enabling the selection of potential high fertility males with higher Linearity and Quantitative factor scores for AI programs, although practical application may vary based on specific breeding goals.

## 5. Conclusions

This study applied factor analysis to dissect complex semen traits in seven chicken breeds. It highlights significant breed-specific differences in semen quality traits and identifies key underlying factors (linearity and quantitative) associated with fertility. Our findings underscore the importance of multivariate analysis in semen quality evaluation and provide actionable insights for enhancing reproductive efficiency in chicken breeding programs.

## Figures and Tables

**Table 1:** Comparison of semen quality traits among seven breeds.

Traits	B	C	R	W	H	T	Y	Pooled-SEM	*p*-Value
VOL, μL	465.67 ^b^	305.41 ^c^	467.00 ^b^	216.79 ^d^	268.97 ^cd^	290.69 ^cd^	558.47 ^a^	76.06	<0.01
CON, 10^8^/mL	18.70 ^c^	28.82 ^a^	22.18 ^bc^	18.50 ^c^	21.41 ^bc^	22.91 ^b^	24.10 ^b^	3.58	<0.01
MOT, %	69.21 ^a^	60.27 ^ab^	69.62 ^a^	48.67 ^cd^	42.64 ^d^	43.15 ^d^	57.85 ^bc^	9.46	<0.01
TSC, ×10^8^	8.97 ^b^	8.92 ^b^	10.15 ^b^	4.07 ^c^	6.08 ^c^	6.42 ^c^	13.47 ^a^	2.28	<0.01
ESC, ×10^8^	6.40 ^a^	5.57 ^a^	7.29 ^a^	2.08 ^b^	2.59 ^b^	2.74 ^b^	7.45 ^a^	1.71	<0.01
pH	7.10 ^cd^	7.08 ^cd^	7.20 ^bc^	7.52 ^a^	7.36 ^ab^	7.30 ^b^	6.99 ^d^	0.18	<0.01
VCL, μm/s	52.09 ^b^	60.11 ^a^	56.24 ^a^	56.80 ^a^	59.01 ^a^	57.03 ^a^	50.79 ^b^	3.40	<0.01
VSL, μm/s	28.39 ^a^	24.12 ^cd^	29.32 ^a^	22.38 ^d^	25.98 ^bc^	27.48 ^ab^	24.28 ^cd^	2.00	<0.01
VAP, μm/s	39.52 ^b^	54.22 ^a^	43.74 ^b^	51.27 ^a^	41.78 ^b^	40.33 ^b^	40.23 ^b^	3.72	<0.01
ALH, μm	5.09 ^b^	5.81 ^a^	5.49 ^a^	5.55 ^a^	5.76 ^a^	5.57 ^a^	4.96 ^b^	0.33	<0.01
BCF, Hz	2.32 ^c^	2.51 ^bc^	2.33 ^c^	2.77 ^ab^	2.70 ^ab^	2.87 ^a^	2.56 ^abc^	0.92	<0.01
LIN, %	54.56 ^a^	41.09 ^c^	52.20 ^a^	40.27 ^c^	44.84 ^b^	48.03 ^b^	47.84 ^b^	3.26	<0.01
WOB, %	75.87 ^c^	89.68 ^a^	77.78 ^c^	89.35 ^a^	79.11 ^bc^	78.97 ^bc^	83.98 ^b^	4.20	<0.01
STR, %	73.01 ^a^	46.61 ^d^	68.22 ^ab^	45.66 ^d^	60.16 ^c^	67.92 ^b^	61.36 ^c^	5.85	<0.01

Note: ^a, b, c, d^ Values within a row with different superscripts differ significantly at *p* < 0.05. W, White Leghorn; B, Barred Plymouth Rock; C, Columbian Plymouth Rock; R, Rhode Island Red; Y, Beijing-You; T, Tibetan; H, Houdan; SEM, standard error of mean; VOL, semen volume; CON, sperm concentration; MOT, sperm motility; TSC, total sperm count; ESC, effective sperm count; pH, semen pH value; VCL, curvilinear velocity; VSL, straight-line velocity; VAP, average path velocity; ALH, amplitude of lateral head; BCF, beat cross frequency; LIN, linearity; WOB, wobble; STR, straightness.

**Table 2:** Variance explained by the first three factors.

Factors	Unrotated		
	Eigenvalue	Percentage of Variance, %	Cumulative Percentage of Variance, %
Factor 1	4.39	31.34	31.34
Factor 2	3.26	23.28	54.62
Factor 3	2.97	21.19	75.82

**Table 3:** Eigenvectors of obtained factors for semen quality traits.

Traits	Factor 1	Factor 2	Factor 3
VSL	**0.92**	0.34	−0.03
LIN	**0.88**	−0.32	0.06
STR	**0.82**	−0.33	−0.03
MOT	**0.51**	0.35	0.47
WOB	**−0.82**	0.30	0.22
BCF	**−0.58**	−0.47	−0.23
VCL	0.00	**0.93**	−0.17
VAP	−0.34	**0.86**	0.00
ALH	0.03	**0.93**	−0.16
TSC	−0.17	−0.11	**0.94**
ESC	0.10	0.11	**0.94**
VOL	0.06	−0.27	**0.78**
CON	−0.38	0.24	**0.56**
pH	−0.05	0.13	**−0.47**

Note: VSL, straight-line velocity; LIN, linearity; STR, straightness; MOT, sperm motility; WOB, wobble; BCF, beat cross frequency; VCL, curvilinear velocity; VAP, average path velocity; ALH, amplitude of lateral head; TSC, total sperm count; CON, sperm concentration; ESC, effective sperm count; VOL, semen volume; pH, semen pH value. Bold indicates that the absolute value of loading is greater than 0.4.

## Data Availability

All relevant data are included in the article.

## Abbreviations

The following abbreviations are used in this manuscript:

AI	artificial insemination
CASA	computer-aided sperm analysis
VOL	semen volume
CON	sperm concentration
MOT	sperm motility
TSC	total sperm count
ESC	effective sperm count
VCL	curvilinear velocity
VSL	straight-line velocity
VAP	average path velocity
LIN	linearity
WOB	wobble
STR	straightness
ALH	amplitude of lateral head
BCF	beat cross frequency
PCA	principal components analysis
FA	factor analysis
ANOVA	one-way analysis of variance
KMO	Kaiser–Meyer–Olkin
